# A Comprehensive Update on Late-Onset Pompe Disease

**DOI:** 10.3390/biom13091279

**Published:** 2023-08-22

**Authors:** Beatrice Labella, Stefano Cotti Piccinelli, Barbara Risi, Filomena Caria, Simona Damioli, Enrica Bertella, Loris Poli, Alessandro Padovani, Massimiliano Filosto

**Affiliations:** 1 Department of Clinical and Experimental Sciences, University of Brescia, 25100 Brescia, Italy; beatrice.labella93@gmail.com (B.L.); stefano.cottipiccinelli@centrocliniconemo.it (S.C.P.); alessandro.padovani@unibs.it (A.P.); 2 Unit of Neurology, ASST Spedali Civili, 25100 Brescia, Italy; loris.poli@asst-spedalicivili.it; 3 NeMO-Brescia Clinical Center for Neuromuscular Diseases, 25064 Brescia, Italy; barbara.risi@centrocliniconemo.it (B.R.); filomena.caria@centrocliniconemo.it (F.C.); simona.damioli@centrocliniconemo.it (S.D.); enrica.bertella@centrocliniconemo.it (E.B.)

**Keywords:** myopathy, late-onset Pompe disease, multi-system disease, diagnosis, enzyme replacement therapy, chaperone, gene therapy

## Abstract

Pompe disease (PD) is an autosomal recessive disorder caused by mutations in the *GAA* gene that lead to a deficiency in the acid alpha-glucosidase enzyme. Two clinical presentations are usually considered, named infantile-onset Pompe disease (IOPD) and late-onset Pompe disease (LOPD), which differ in age of onset, organ involvement, and severity of disease. Assessment of acid alpha-glucosidase activity on a dried blood spot is the first-line screening test, which needs to be confirmed by genetic analysis in case of suspected deficiency. LOPD is a multi-system disease, thus requiring a multidisciplinary approach for efficacious management. Enzyme replacement therapy (ERT), which was introduced over 15 years ago, changes the natural progression of the disease. However, it has limitations, including a reduction in efficacy over time and heterogeneous therapeutic responses among patients. Novel therapeutic approaches, such as gene therapy, are currently under study. We provide a comprehensive review of diagnostic advances in LOPD and a critical discussion about the advantages and limitations of current and future treatments.

## 1. Introduction

Glycogen storage disease type II (GSDII), also named Pompe disease (PD), is an autosomal recessive disorder caused by a deficiency in the acid alpha-glucosidase enzyme (GAA), which is responsible for the hydrolysis of glycogen to glucose in the lysosome [1,2].

In the past, the prevalence and expected frequency of PD were reported to be around 1 in 40,000 births [3,4,5,6]. However, thanks to the implementation of newborn screening (NBS), the prevalance seems to be much higher, ranging from 1.0 per 4447 to 1.0 per 37,094, with differences between countries related to ethnic factors and screening tools [7,8,9,10,11,12,13,14,15,16,17,18]. Data from the main epidemiological studies are detailed in Table 1.

The *GAA* gene (OMIM *606800) is located on chromosome 17q25 and it is the only gene associated with Pompe disease (OMIM #232300) in the online Mendelian Inheritance in Man Database [19,20]. It encodes a 110 kDa precursor polypeptide that undergoes several post-translational modifications in the rough endoplasmic reticulum (ER) [19,20,21]. After N-glycosylation and proper folding in the ER, the enzyme is transported to the Golgi, where it acquires mannose 6-phosphate (M6P), which is required to target the lysosome via the cation-independent mannose 6-phosphate receptor (CI-M6P) pathway [21,22,23,24,25,26]. CI-M6P receptors are transmembrane glycoproteins located mostly in the trans-Golgi and endosomal compartments, but also at low levels at the cell surface [21]. CI-M6P receptor cycling over the cell surface is crucial for enzyme replacement therapy (ERT) because it represents the key to enzyme access in the cell [21,27].

*GAA*, contained in a vesicle that pinches off from the Golgi to the endosomes, undergoes a series of proteolytic and N-glycan processing steps that are necessary for its activation [21,22,28,29,30]. In patients affected by PD, the defective enzyme is not able to catalyze hydrolysis of the α-1,4 and α-1,6-glucosidic bonds in glycogen, thus leading to increased glycogen storage in lysosomes, which is responsible for lysosomal dysfunction, enlargement, and finally rupture [28,31]. However, the pathogenesis of PD is not simply related to the presence of damaged lysosomes; defective autophagy, dysregulation of lysosome-based signaling pathways (i.e., the nutrient-sensitive mammalian target of rapamycin complex 1—mTORC1), oxidative stress, and mitochondrial abnormalities take part in the pathogenic cascade and contribute to altering muscle architecture with displacement of the myofibrils [19,28,31].

**Table 1:** Data from main epidemiological studies on Pompe disease.

Methods and Source	Period of Study	Expected Frequency of GSDII Cases	Population and Country	Reference
Estimation of the frequency of PD by determining carrier status in a randomly selected healthy population for seven of the most common mutations.	1998	1.0 per 40,000	N = 928 healthy individuals Five different race groups. Mostly aged between 20 and 50 y.o. *USA*	Martiniuk F et al. Am J Med Genet. 1998 [4]
Calculation of the frequency of PD by screening a random sample of newborns for three frequent mutations in the acid alpha-glucosidase gene (IVS1(-13T®G), 525delT and delexon18). The screening was performed on DNA extracted from over 3000 Guthrie cards from Dutch neonates.	1999	1.0 per 40,000 (1.0 per 138,000 for infantile GSDII; 1.0 per 57,000 for adult GSDII)	N = over 3000 Guthrie cards from Dutch neonates *Netherlands*	Ausems M. et al. Eur J Hum Genet. 1999 [5]
Calculation of birth prevalence of lysosomal storage diseases (LSDs) based on records from the laboratories of the clinical genetic centers involved in the post- and prenatal diagnosis of LSDs.	1970–1996	Birth prevalence of 2.0 per 100,000 live births	N = 963 enzymatically confirmed cases *Netherlands*	Poorthuis BJ et al. Hum Genet. 1999 [3]
Calculation of birth prevalence of each LSD by dividing the total number of diagnosed cases (post- and prenatal diagnosis) by the total number of live births that occurred between the years of birth of the older and younger patients (birth period).	1940–1999	Birth prevalence of 0.17 per 100,000	Data from total N of live births from Instituto Nacional de Estatistica, Porto *North Portugal*	Pinto R et al. Eur J Hum Genet. 2004 [6]
Frequency of PD assessed by newborn screening program.	2010	1.0 per 8684	N = 34,736 newborns screened *Austria*	Mechtler TP et al. Lancet. 2012 [15]
Frequency of PD assessed by newborn screening program.	2011	1.0 per 4447	N = 40,024 newborns screened *Hungary*	Wittmann J et al. JIMD Rep. 2012 [12]
Frequency of PD assessed by newborn screening program.	2013–2016	1.0 per 34,401	N = 103,204 newborns screened *Japan*	Momosaki K et al. Hum Genet. 2019 [18]
Frequency of PD assessed by newborn screening program.	2016–2019	1.0 per 16,095	N = 531,139 newborns screened *Pennsylvania, USA*	Ficicioglu C et al. Int J Neonatal Screen. 2020 [8]
Frequency of PD assessed by newborn screening program.	2014–2019	1.0 per 23,596	N = 684,290 newborns screened *Illinois, USA*	Burton BK et al. Int J Neonatal Screen. 2020 [14]
Frequency of PD assessed by newborn screening program.	2018–2019	1.0 per 25,200	N = 453,152 screened newborns *California, USA*	Tang H et al. Int J Neonatal Screen. 2020 [9]
Frequency of PD assessed by newborn screening program.	2013–2018	1.0 per 10,152	N = 476,000 screened newborns *Missouri, USA*	Klug TL et al. Int J Neonatal Screen. 2020 [10]
Frequency of PD assessed by newborn screening program.	2019	1.0 per 19,777	N = 59,332 screened newborns *Georgia, USA*	Hall PL et al. Int J Neonatal Screen. 2020 [16]
Frequency of PD assessed by newborn screening program.	2013–2020	1.0 per 37,094	N = 296,759 newborns screened *Japan*	Sawada T et al. Orphanet J Rare Dis. 2021 [7]
Frequency of PD assessed by newborn screening program.	2015–2022	1.0 per 18,432	N = 206,741 screened newborns *Italy*	Gragnaniello V et al. Mol Genet Metab Rep. 2022 [11]

## 2. Phenotypes and Phenotypic Heterogeneity

PD represents a spectrum of phenotypes that differ in age of onset, organ involvement, severity of disease, and rate of progression [1,2]. PD phenotypes are named according to age at onset, with infantile, childhood, juvenile, and adult forms [32,33,34,35,36,37,38]. Infantile-onset Pompe disease encompasses classical and non-classical infantile forms [32,33]. The classical infantile form usually presents in the first days or weeks of life, and it is associated with cardiomegaly and hypertrophic cardiomyopathy [32,33]. Skeletal impairment manifests with feeding difficulties, hypotonia, and respiratory distress [32,33]. Death occurs within the first two years of life due to progressive cardiac and respiratory insufficiency [32,33]. The latter is mainly caused by weakness of the diaphragmatic and abdominal wall muscles, especially the internal obliques and rectus abdominis [32,33]. In this review, the acronym “IOPD” refers to the classical infantile form.

The term “non-classical infantile form” is applied to infants under 1 year presenting with less severe cardiac involvement and milder motor symptoms, allowing longer survival and better motor development [32,33].

The term “LOPD” is applied to patients with an onset between 12 months and adulthood (childhood, juvenile, and adult forms) [34,35,36,37,38,39]. The LOPD phenotype may be extremely variable, encompassing cases with mild muscle involvement to those with severe chronic respiratory failure and marked limb-girdle and axial weakness [34,35,36,37,38,39].

In common use, the term “LOPD” is applied also to asymptomatic patients who harbor LOPD mutations in the *GAA* gene diagnosed because of family history, elevated serum creatine kinase levels, or NBS, and whose clinical expression is not predictable at diagnosis [35,36,37,38].

The heterogeneous severity of the disease is related to the residual activity of the GAA enzyme in tissues [20,40]. Some *GAA* mutations are particularly deleterious and cause a complete lack of enzyme, while other less severe mutations allow a certain residual enzyme activity [36,41,42,43,44,45]. Known mutations in the *GAA* gene are reported in the “Pompe Disease *GAA* variant database” (http://www.pompevariantdatabase.nl, accessed on 1 May 2023). Details of the most common variants are provided in Table 2.

In the genotype-phenotype report from the Pompe Registry published in 2019, the non-sense variant c.2560C>T is frequently reported in IOPD, tracing its origins from Northern Africa and being diffuse in North and Latin America due to the slave trade [42]. The missense c.1935C>A variant is the most frequent variant in IOPD patients from the Asia-Pacific area and is frequently associated with the pseudo-deficiency variant c.1726G>A [42].

The c.-32–13T>G variant is typically associated with the LOPD phenotype spectrum [45,46,47]. Earlier onset in patients with the c.-32–13T>G variant is possibly related to the concomitant presence of more deleterious variants in *GAA*, such as c.510C>T [46,47].

Among the most frequent variants, the c.525delT variant and exon 18 deletion can be associated with either IOPD or LOPD, depending partially on the nature of the other allele [42]. The presence of a variant close to the splice site can affect splicing and may be predictive of LOPD, while splice site variants, non-sense variants, deletions, and insertions are often associated with IOPD [42].

However, genetics cannot alone determine the phenotypic variability in PD and cannot explain, for example, the variability in terms of age of onset, severity, and distribution often observed in individuals carrying the same mutations [48,49,50]. Variants in modifier genes have been proposed to influence these characteristics, such as *ACE* and *ACTN3* polymorphisms that are known to affect fiber type composition and muscle properties [49]. Nevertheless, a recent clinical trial with 24 families did not show clear influence of ACE I/D polymorphism on clinical presentation or response to ERT, as patients with discordant *ACE* genotypes had similar disease courses while siblings with the same *ACE* genotype presented with different disease courses [51].

Indeed, more studies are needed to investigate the possible influence on clinical variability of epigenetic and trans-acting factors that may regulate lysosomal or skeletal muscle homeostasis [48,50].

## 3. Clinical Assessment and Diagnostic Tools

Family history of muscle diseases, age of onset, and course of disease should always be carefully assessed. Progressive limb-girdle weakness is one of the most frequent features reported by patients with adult-onset LOPD and often the main reason for clinician evaluation [52]. However, red flags include isolated respiratory muscle involvement and isolated elevated serum creatine kinase levels, found in around 2.5% of patients in population studies [52].

Neurological examination should include evaluation of trunk muscles that can be affected early in LOPD, even though their involvement is difficult to clinically assess because the ability to rise from the supine position or raise the trunk from the prone position requires activation of different muscle groups and may be influenced by osteoarticular conditions or respiratory dysfunction [53]. The presence of scoliosis may suggest weakness of trunk muscles [53].

Tongue weakness can be an early sign of disease and should always be evaluated, as it may be useful in differentiating LOPD from other forms of myopathy [54]. Clinical assessment of tongue weakness can be later confirmed through magnetic resonance imaging (MRI) using the so-called “bright tongue sign,” which unfortunately may be missed or unreported by non-expert radiologists [54,55,56,57].

Laboratory tests usually reveal a mild increase in creatine kinase (CK) levels in LOPD, although these can sometimes be normal [58,59,60,61,62]. Elevation of plasma cardiac troponin T (cTnT) levels, which reflects skeletal muscle damage, can also be found and may erroneously lead to the diagnosis of myocardial infarction or injury [63]. Commonly, elevated alanine aminotransferase (ALT) and aspartate aminotransferase (AST) levels are found and should be considered red flags for suspicion of metabolic myopathies [64]. However, all of these laboratory findings are nonspecific and have limited prognostic value.

Electromyography (EMG), other than showing a myopathic pattern with spontaneous activity, may detect complex repetitive discharges and electrical myotonia in axial muscles, which always raises suspicion of the LOPD phenotype spectrum [65,66]. Rare cases of neurogenic pattern are reported in the literature, leading to misdiagnosis as spinal muscular atrophy [67].

Muscle MRI represents a promising diagnostic tool for early diagnosis and follow-up in the LOPD phenotype spectrum [68,69,70]. The most typical MRI pattern is systematic axial muscle involvement, especially in trunk extensors [71]. These abnormalities may occur even in the pre-symptomatic stage of disease, possibly driving clinical suspicion [71]. Paravertebral atrophy is not specific and may be detected in other inherited or acquired myopathies, but its association with abdominal belt weakness is not commonly observed in other diseases [72,73,74]. Mild or moderate involvement of the adductor magnus muscle may be evident in the early stages of disease, followed by the semimembranosus, and later, the long head of the biceps femoris and the semitendinosus [75,76]. Fat replacement in knee extensors is usually detected in more advanced stages of disease, with selective sparing of the rectus femoris, sartorius, and gracilis muscles; this pattern can be useful in differential diagnosis from other muscular diseases, such as dystrophies [75,76]. In the upper limbs, involvement of the trapezius inferior, rhomboid, and subscapularis muscles with later moderate damage of the serratus anterior, deltoid, and supraspinatus muscles is usually described [37,71]. Diaphragmatic MRI abnormalities may be present, even when functional pulmonary tests are still within normal range [77,78,79].

**Figure 1:**
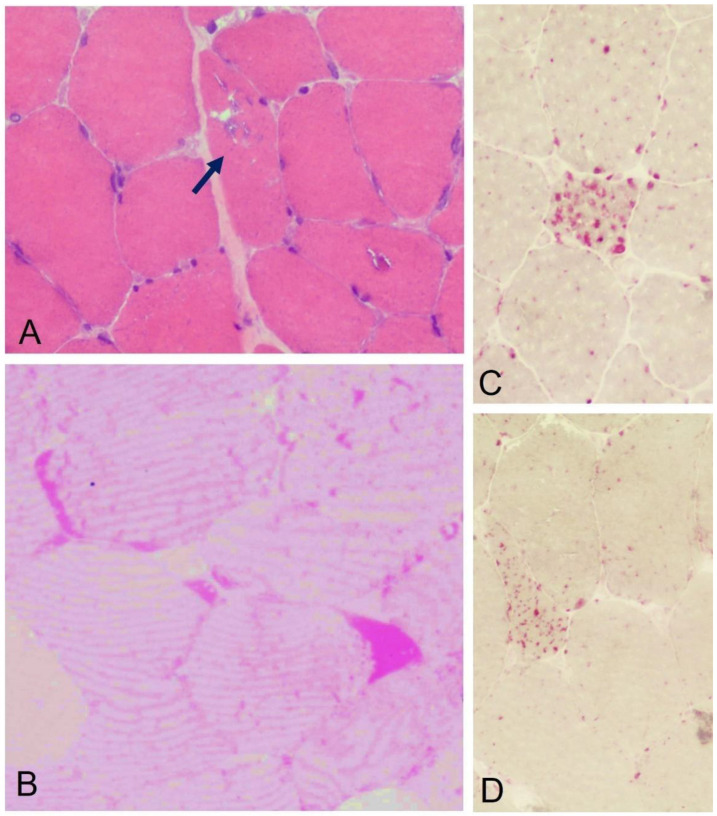
Quadriceps muscle biopsy in patients with LOPD. (**A**) Hematoxylin-eosin staining showing the presence of intracytoplasmic vacuolization with a “rimmed” appearance (arrow). (**B**) Periodic acid-Schiff (PAS) staining on semi-fine section showing the presence of abnormal accumulation of intrafibral glycogen. (**C**,**D**) Acid phosphatase staining showing the presence of lysosomal activation in scattered fibers.

Muscle biopsy may be performed using standard procedures in histological and histochemical studies, including Periodic acid-Schiff (PAS) staining for identifying glycogen accumulation and acid phosphatase staining as a marker of lysosomal abnormalities [80,81]. The typical pathological pattern is characterized by vacuolar myopathy with glycogen storage and increased acid phosphatase reactivity (Figure 1) [80,81]. Fiber atrophy is more evident in IOPD and childhood forms, with type 1 and type 2 fibers equally involved [80,81]. The presence of internal nuclei and necrosis is also reported in adult cases [81]. The number of vacuoles and amount of glycogen accumulation are variable and usually more prominent in IOPD than in adult forms, in which histological studies may also show mild involvement or, in some cases, normal appearance [81,82]. In a French study of 21 patients with LOPD, muscle biopsy was negative in 30%, despite confirmation by a positive blood-based enzyme assay [83]. Correct conservation of the sample and optimal fixation, embedding, and staining methods may play a role, and PAS on a semi-thin section has been found to be more reliable, allowing better visualization of glycogen-filled vacuoles, regardless of their size [81,84,85]. It has also been suggested that detection of large lipofuscin inclusions within the area of autophagic accumulation using high-resolution light microscopy can be suggestive of PD [86]. Genetic analysis is considered the gold standard for a definitive diagnosis and can be performed on blood, cheek swab, muscle tissue, or saliva samples [87,88]. While in the past the diagnostic process always included muscle biopsy and enzyme activity evaluation followed by targeted genetic studies, technological advances in genetics have greatly changed clinical practice [87,88]. The availability of NGS panels that allow rapid analysis of a large number of genes has meant that, often, genetic study has become the first diagnostic step following clinical suspicion [87,88]. However, if variants of undefined pathogenetic significance are found, muscle biopsy and biochemical assays remain of great importance in defining their role in causing disease. A biochemical assay of GAA activity can be performed in mixed leukocytes, fibroblasts, or muscle tissue [40,58,88,89,90]. In general, GAA activity is extremely reduced in IOPD patients (<1%), while LOPD patients have 2–30% of normal GAA activity [40,91].

In many centers, faced with suspicion of LOPD based on clinical red flags such as limb-girdle or axial weakness or elevated serum creatine kinase levels, screening is performed by assaying acid alpha-glucosidase activity on a dried blood spot (DBS) via tandem mass spectrometry or fluorometry [92,93]. It is frequently a first-line test because it has the advantages of being easy to perform, inexpensive, minimally invasive, and able to provide rapid results [92,93,94,95,96,97]. In the past, discrepancies in GAA activity could be related to isozymes (e.g., renal isozyme or isoenzyme maltase-glucoamylase) that showed activity at the (acidic) pH used to measure activity, interfering with the results of clinical assays [95,96]. For this reason, newer blood assays use acarbose to competitively inhibit maltase-glucoamylase [90,96,97,98]. Also, incorrect blood spotting and the combination of humidity and heat caused by insufficient drying or inappropriate shipping can interfere with enzyme stability and, consequently, the assessment of GAA levels [98]. Rarely, positive DBS with no pathogenic variant may also be related to pseudodeficiency alleles [40,99,100]. As a screening test for LOPD, quantification of PAS-positive lymphocytes in peripheral blood has recently demonstrated its usefulness [101]. Moreover, a decrease in the percentage of PAS-positive lymphocytes in a small subgroup of LOPD patients suggests that it could be useful as a therapy response biomarker [101]. Urinary glucose tetrasaccharide (Glc4) could be used as a second-tier test after positive DBS, and its concentration seems to correlate with age of symptom onset, with more elevated levels in IOPD patients [102,103,104,105]. However, elevated urinary tetrasaccharide glucose levels are not specific to PD and may also be related to urinary infections, acute pancreatitis, muscular trauma, and some cancers [102]. Defining reference intervals is still being debated, as some affected patients can have normal Glc4 excretion in the early phases of disease [102,104]. A summary of the main clinical findings in LOPD is reported in Table 3.

## 4. Multisystem Involvement and Indications for Follow-up and Management

A multidisciplinary approach for adequate management and follow-up is fundamental, as respiratory dysfunction and cardiac involvement impact the survival of patients [37]. Practical recommendations for pulmonary assessment include an anamnestic questionnaire, clinical examination, and pulmonary functioning tests [106]. If patients complain of morning headaches or excessive daily sleepiness, an overnight cardiorespiratory polysomnography is recommended to rule out nocturnal hypoventilation and the development of chronic daytime hypercapnia [107]. To assess respiratory failure, it is important to measure forced vital capacity (FVC) both in seated and supine positions: a drop of over 25% in the percentage of predicted FVC upon changing posture from the upright to the supine position is suggestive of diaphragmatic weakness [108,109,110]. As weakness of the diaphragm may be an early sign and evolves independently to skeletal muscle status, close follow-up is recommended, and pulmonary function should be monitored routinely once or twice per year [106,111]. Clinical indications for the introduction of non-invasive or invasive ventilation are detailed in the American Thoracic Society and European Respiratory Society guidelines and in the paper by Boentert et al. [106,112].

A standard cardiac assessment, including clinical history, physical examination, a 12-lead electrocardiogram, and echocardiography, is required for each patient and may be repeated every 1–2 years in case of normal findings [113]. Severe cardiac involvement is present in IOPD and may lead to left ventricular (LV) outflow obstruction resulting in fatal cardiac failure [114,115]. Right ventricular systolic function is usually preserved in LOPD, while diastolic LV dysfunction has been rarely reported [116,117,118,119,120]. Atrial enlargement may be detected as a consequence of poor ventricular compliance and diastolic dysfunction [119]. A Holter electrocardiogram should be performed in order to rule out rhythm disturbances such as Wolff–Parkinson–White syndrome, often in association with a short PR interval or second-degree atrioventricular block [120,121,122]. However, cardiac involvement in LOPD has been recently questioned, as prevalence may be biased by the lack of data on established cardiovascular risk factors (e.g., obesity, hypertension, dyslipidemia, diabetes mellitus) among patients in most cohort studies [123]. A periodic neurological follow-up in LOPD should include clinical evaluation by assessment of muscle strength and basic functional tests (e.g., Gowers’ maneuver), combined with laboratory tests (e.g., CK) and muscle MRI [124]. Cerebral MRI with sequences for intracranial and extracranial large vessel study should be included at baseline and repeated every two years, as cerebrovascular manifestations (e.g., aneurysms, vertebrobasilar dolichoectasia, dilatative arteriopathy) have been reported [125,126,127,128]. If an intracranial aneurysm is identified, the first step is assessing risk factor stratification for rupture depending on aneurysm-related (e.g., size or shape) and patient-related (e.g., age, cardiovascular risk factors) findings [129]. If a high-risk profile is defined, endovascular treatment is indicated [129]. Evidence of small vessel disease in LOPD is reported in the literature, although data corrected for baseline cardiovascular risk factors are few [130]. Differently, IOPD patients may present with diffuse leukoencephalopathy related to glycogen storage on the CNS, affecting mostly periventricular white matter and centrum semiovale areas, associated with cognitive decline [131,132]. The impact on cognitive profile seems to be less severe in LOPD, although functional disruption of neuronal networks involved in executive functioning, planning, and abstract reasoning was found even without grey matter atrophy in small cohort studies [130,133].

Other organ involvement is usually assessed according to the patient’s referred symptoms. For example, degeneration of bones and joints as a consequence of muscle weakness is a frequent finding, as scoliosis, kyphosis, and hyperlordosis are usually linked to abdominal and hip extensor weakness [37]. Rarely, more severe phenotypes may present with rigid spine syndrome, characterized by limited flexion of the cervical and dorsolumbar spines, or bent spine syndrome in which weakness of extensor spine muscles causes a pathological anterior curvature of the thoracolumbar tract [37,134]. Routine spine and femoral radiography with bone densitometry are recommended periodically because they may unveil asymptomatic and atraumatic vertebral fractures that are not always related to significant impairment of bone mass [37,134].

## 5. Future Perspectives in Monitoring LOPD Symptoms

While Glc4 was initially applied to monitor the response to ERT with only mild results, more recent studies have focused on serum microRNA profiles as potential biomarkers of disease [135,136]. Elevated levels of so-called dystromirs (miR-1-3p, miR-133a-3p, and miR-206), which are microRNAs involved in muscle regeneration, have been found in PD and other neuromuscular diseases [136]. In a sample of 35 patients with LOPD, miR-206 levels were significantly higher in serum samples of symptomatic patients compared to asymptomatic patients, suggesting that this microRNA could be used to follow up presymptomatic patients and guide the timing of treatment [136]. Correlations between serum levels and severity of disease were also observed in the plasma samples of 52 patients, where miR-133a levels were higher in infantile-onset patients than in late-onset subjects [137]. Although promising, these recent data are applied only in research studies for now and need further confirmation.

Meanwhile, in the past 10 years, muscle MRI techniques have developed and emerged as an interesting tool not only for diagnosis but also for follow-up in LOPD. A recent study performed with conventional T1-TSE sequences and assessment of fat replacement with Mercuri scoring suggested the stabilization of the fat fraction of the psoas muscle under ERT, yet several follow-up MRI studies were able to detect the slow progression of the skeletal muscle fat fraction by using more sensitive sequences such as water/fat (Dixon) [138,139,140]. As fatty muscle replacement can occur before clinical weakness, and mildly affected muscles seem to respond better to ERT than severely involved ones, MRI studies may help to decide when to start ERT in clinically asymptomatic patients [141].

Although muscle MRI is the gold standard in imaging evaluation in order to follow up and evaluate treatment response, ultrasonography could be an interesting alternative if muscle MRI is not available, as a recent study suggested it to be efficient in evaluating diaphragm dysfunction [142].

## 6. Treatment Approaches

The main current and future therapeutic approaches are summarized in Table 4.

### 6.1. Enzyme Replacement Therapy (ERT)

Disease severity increases with disease duration, and past observational studies have shown that, every year from diagnosis, the odds for wheelchair use increase by 13% and the odds for respiratory support increase by 8% [143]. In 2006, perspectives changed thanks to the approval of ERT based on the intravenous infusion of a recombinant human GAA (rhGAA) precursor enzyme for IOPD [21,144]. The uptake of rhGAA from the bloodstream into the cells is driven by the M6P receptors, which later target rhGAA to the lysosomes, where it can be turned into the active form [19,21,144].

In IOPD patients, rhGAA (alglucosidase alfa) clearly improved survival with a decreased risk of invasive ventilation and preserved cardiac function, yet a therapeutic problem emerged in the so-called IOPD CRIM-negative patients, who have two deleterious *GAA* mutations and are completely unable to make native enzyme [145]. IOPD CRIM-negative patients had poorer clinical outcomes because of their immunological response to rhGAA, so immunomodulatory therapy was recommended in order to prevent this side effect [145,146,147].

After promising results in IOPD patients, a randomized, placebo-controlled trial with alglucosidase alfa (LOTS trial) was conducted with 90 LOPD patients (8 years old or older; mean age at first infusion: 45.3 for the treated group and 42.6 for the placebo group) [148]. Patients received biweekly intravenous alglucosidase alfa (20 mg per kilogram of body weight) or placebo for 78 weeks at eight centers in the United States and Europe, and the primary endpoints (improvement of distance walked during a 6-min walk test and percentage of predicted FVC) were reached [148]. The open-label extension LOTS trial and several other studies confirmed the stabilization of motor and pulmonary function, which correlated with reduced lysosomal glycogen levels after ERT in the EMBASSY study [148,149,150,151,152,153,154].

Interestingly, the reduction in vacuolated fibers and PAS-positive material in muscle biopsies after ERT was more marked in less affected fibers and in smaller PAS-positive collections, thus underscoring that the efficacy of ERT was greater when initiated in the early stages of disease [155]. However, according to the European consensus, clinical or instrumental manifestation of PD is required to start treatment, which can be later stopped only in cases of severe infusion-associated reactions, immunogenicity, or loss of efficacy for more than two years [156]. Follow-up studies revealed significant variability in response to therapy [157,158,159,160,161,162,163,164]. Usually, LOPD patients very rarely develop high titers of neutralizing anti-rhGAA antibodies and, therefore, immunogenicity would not explain the variable therapeutic response [155,157,158,159,160,161,162,163]. A mild or moderate improvement in LOPD patients may be observed during the first 2 to 3 years of treatment, followed later by a plateau or slight decline [157,158,159]. A recent 10-year follow-up study confirmed this trend and underlined the variability in duration of the plateau phase among LOPD patients [164]. Younger age and better clinical status for supine FVC or muscle strength were initially identified as good prognostic factors for response to treatment, yet long-term follow-up in treated patients did not confirm this evidence [152,157,163]. The reasons for the progressive decline are still unclear. Several hypotheses have been proposed, including possible limited biodistribution of rhGAA and reduced intracellular uptake due to low expression of the CI-M6P receptors on skeletal muscle cells [165].

One of the first options to overcome limited therapeutic efficacy was to design novel rhGAAs, such as avalglucosidase, produced by chemical conjugation of an oligosaccharide harboring bis-M6P residues onto recombinant human GAA via oxime chemistry, thus increasing affinity for the CI-M6P receptors and achieving larger distribution in systemic tissues [166]. After the first positive results of avalglucosidase in the NEO1 trial, a recent randomized, double-blind trial tested its efficacy and safety compared to alglucosidase (COMET trial) [167,168,169]. Participants in the COMET trial received intravenous infusions of alglucosidase alfa or avalglucosidase alfa (20 mg/kg) every 2 weeks [169]. The primary objective for efficacy, non-inferiority in respiratory function (as measured by upright FVC% predicted), was met and far exceeded the predefined margin [169]. Although the primary objective was met and the safety profile seemed more favorable than that of alglucosidase alfa, superiority testing did not reach statistical significance [169]. Avalglucosidase was approved for treatment of LOPD in 2021 and the recommended dose is 20 mg/kg body weight, administered once every 2 weeks.

A weight-tiered mg/kg dosing regimen of avalglucosidase alfa in IOPD patients (40 mg/kg every 2 weeks <30 kg) has been defined, supported by data from a model-informed extrapolation approach [170]. In IOPD, a 6-month primary analysis of the mini-COMET study recently showed a trend toward improvement or stabilization of clinical outcomes and a good safety profile, with better results for patients treated with avalglucosidase alfa 40 mg/kg every 2 weeks [171]. The Baby-COMET trial to assess efficacy in naïve IOPD patients is still recruiting.

In order to optimize ERT efficacy, proper exercise training and nutrition should be also considered [172,173,174]. A small cohort of LOPD patients showed improvement in laboratory tests (e.g., creatine kinase, lactate dehydrogenase) and exercise tolerance after introduction of personalized sessions of training and/or a high-protein diet [173]. A randomized, controlled study evaluating lifestyle interventions in IOPD and childhood-onset LOPD (median age: 10 years old) showed improvement in muscle strength, core stability, muscle function, quality of life, and fatigue after a tailored 12-week program [174].

### 6.2. Pharmacological Chaperone Therapy

Pharmacological chaperone therapy (PCT), first experimented in treatment of Fabry disease, is based on the concept that small-molecule ligands may act by blocking conformational fluctuations of a partially misfolded protein, thereby facilitating its trafficking into organelles [175,176]. In 2007, the action of the chaperone deoxynojirimycin (DNJ) and its derivative, N-butyldeoxynojirimycin (NB-DNJ), on the fibroblasts of Pompe patients showed a significant increase in GAA activity in fibroblasts carrying particular mutations [177,178]. However, the responsiveness to PCT alone seemed to be influenced by the location and nature of the amino acid substitutions, as an in vitro study showed that DNJ had no effect on 17 variants among the 76 tested mutant forms of GAA [176].

While facing bioavailability and pharmacodynamics limits of rhGAA, one strategy to improve these aspects was to use enzyme stabilizers to modulate GAA enzymatic activity. In fact, co-incubation of Pompe fibroblasts with recombinant human α-glucosidase and NB-DNJ revealed increased activity of GAA, which was later confirmed in animal model studies [179,180]. The results of the first clinical trial with combination therapy were published in 2014, comparing rhGAA ERT plus NB-DNJ chaperone (miglustat) to ERT alone in 13 patients with PD (3 infantile-onset and 10 late-onset) [181]. After a 2-month assessment of ERT alone, patients were treated with combination therapy for 12 months, then switched again to ERT alone and reevaluated after 2 months [181]. Increased and prolonged GAA activity with combination therapy compared to ERT alone was assessed by DBS, yet no clinical outcomes were analyzed because of the limitations of the study protocol [181].

Later, an open-label trial with different doses of chaperone (from 50 to 600 mg) confirmed this evidence, showing a 1.2- to 2.8-fold increase in total GAA activity after administration of PCT in 25 patients previously treated with ERT alone [182]. Recently, a phase 3 trial combining cipaglucosidase alfa, a new rhGAA with enhanced glycosylation for improved cellular uptake and processing, plus miglustat (n = 85) compared to alglucosidase alfa plus placebo (PROPEL study) was published in 2022 [183]. Although the primary endpoint (changing 6MWT) did not reach statistical superiority over alglucosidase, a meaningful improvement in motor and respiratory functions at 52 weeks was observed with cipaglucosidase alfa plus miglustat compared to alglucosidase alfa plus placebo [183]. These promising results led to the approval on 27 March 2023 of cipaglucosidase alfa as a long-term ERT used in combination with miglustat for the treatment of adults with LOPD in the EU [184].

To date, the advantages of combination therapy include possible blood–brain barrier crossing and better bioavailability of rhGAA, yet further data from the open-label phase of the PROPEL trial are needed to better define therapy response and clinical outcomes.

### 6.3. Gene Therapy

The risk of immunogenicity complications and the pharmacodynamics problems related to ERT have stimulated the development of different therapeutic approaches. This is the reason why gene therapy, predominantly adeno-associated viral (AAV) gene therapy for in vivo applications and lentiviral (LV) gene therapy for ex vivo applications, has gained much interest in the last few years.

#### 6.3.1. AAV Vector-Mediated Gene Therapy in Pompe Disease

AAV vectors contain an icosahedral capsid that is serotype- and tissue-specific, depending on which receptor or coreceptor is used to enter tissue cells [185,186]. Clearly, the first experimental studies focused on serotypes endowed with muscle tropism, such as AAV9, AAV6, and AAV1 [187,188]. In an animal model, injection of theAAV2/6 vector in the gastrocnemius led to an increase in GAA activity in the injected muscle, even though no significant cross-correction was observed in the contralateral gastrocnemius, liver, or heart [187]. Limited results were also reported after intramuscular injection of the AAV9 vector at different stages of disease in a murine model, which showed that, despite high levels of AAV9-*GAA* tissue transduction and clearance of glycogen from myofibers, advanced-stage mice showed no improvement in motor function [188]. As respiratory dysfunction is one of the most severe comorbidities in PD, local intradiaphragmatic AAV delivery was piloted in preclinical studies and in a phase I/II AAV trial conducted with five ventilator-dependent children [189]. Although mild improvement in ventilatory endurance was observed, no significant change in maximal inspiratory pressure was identified and all patients developed variable Ig antibody responses to the AAV capsid proteins [189]. As similar results were later reported with four other patients, researchers lost interest in intramuscular AAV administration due to its poor rate of correction and they looked for a possible systemic route of administration [190,191]. In a murine model, intravenous AAV vector encoding the human GAA protein under control of a muscle-restricted promoter/enhancer element (AT845) led to functional improvement and glycogen clearance in skeletal and cardiac muscles, with accumulation of active enzyme in the muscle tissue at supraphysiological levels [191]. However, the safety outcome showed dose-dependent multi-organ toxicity in cynomolgus macaques, probably related to a xenogeneic immune response [191].

To improve CNS glycogen clearance, preclinical and animal studies have explored spinal, intrathecal, or intracerebroventricular delivery of AAV-*GAA* [192,193,194]. Intracerebroventricular injection of AAV-*GAA* transduced in the cortex and cerebellum decreased astrogliosis and improved myelination in a Pompe mouse model, yet a poor muscle glycogen storage response was observed [194].

A more recent study with a murine model applied intravenous administration of an AAV vector containing a regulatory cassette to drive *GAA* expression (AAV-PHP.B- chicken b-actin [CB]-h*GAA*) in the heart, skeletal muscles, and CNS [195]. Although reduced efficacy in copy numbers compared to CNS tissues was observed, therapeutic levels of GAA activity were achieved in muscle tissues with no adverse immune effects [195]. Taking inspiration from studies aimed at treating hemophilia B, liver-AAV vectors have been evaluated to correct GAA activity across the entire body, with potential benefits also in inducing antigen-specific immunological tolerance [196,197,198]. Administration of an adenoviral vector containing the CMV enhancer/promoter to drive *GAA* liver expression showed increased hepatic production of the human GAA precursor enzyme with consequently higher levels of GAA activity in plasma [185]. This approach was based on the idea that saturation of the receptor-mediated lysosomal targeting mechanism by over-expression of *GAA* in the liver would lead to secretion of the enzyme precursor into the circulatory system and increase its distribution to other tissues [185]. Liver-AAV vector treatment was associated with great improvement in glycogen clearance in the heart and variable responses in muscles, suggesting the presence of a threshold of circulating GAA activity required to achieve glycogen clearance in different tissues [198,199].

In a novel *GAA* knock-out model with a severe respiratory phenotype, systemic early administration of the AAV-sec *GAA* vector using a liver muscle promoter not only cleared pathological glycogen storage in the spinal cord but also respiratory impairment, which was at least partially influenced by neuroinflammation [200]. To further increase secretion of GAA, the use of a chimeric enzyme containing an alternative signal peptide was also applied, with mildly positive results [201].

Combined gene and enzyme replacement therapy has been studied in a murine model, based on the immunomodulatory effects of liver-AAV gene therapy [202]. After a first phase of administration of ERT or the AAV vector to compare their efficacy in glycogen clearance showed similar results, groups were treated with the vector alone or combined with the ERT to define a minimum dose of the AAV vector needed to induce immunotolerance [202]. However, further studies are required to evaluate the long-term effects and determine if a satisfactory response could be induced in long-treated ERT patients. Unfortunately, immunogenicity is still a problem for AAV vector therapies [203,204,205]. Although they suggest possible immunotolerance, the results of preclinical studies of AAV-liver gene therapy are variable and influenced by serotype selection, vector composition, dosing, and choice of immune modulation [198]. Furthermore, a large quantity of AAV vector would be required to treat Pompe patients because of hepatocyte-restricted expression, possibly exposing them to risk of immune responses against the transgene product and/or AAV capsid.

A recent study tried to assess the minimum effective dose of the administered AAV8 vector, which was set in preclinical studies at around 2 × 10^11^ vector genomes (vg)/kg body weight [186]. Although a good liver response in GAA activity was seen at this dose, a 4-fold higher dose was required to significantly achieve GAA activity in the heart, diaphragm, and quadriceps [186]. It has also been noted that GAA biochemical correction in animal models differs according to the age of the treated animals, as shown by comparing the effects of gene therapy in mice with PD aged 2 weeks or 2 months, raising the need of evaluate dose adjustment [206]. Biochemical correction was far superior in older mice with decreased left ventricle mass, decreased breathing frequency, and increased wire hang latency [206]. The latter two improvements were greater in females than in males despite a lower trend of correction of GAA activity, suggesting unknown sex-related differences in response to therapy [206]. In this regard, a recent study evaluating gene therapy response in a female mouse model suggested improvement of GAA activity after administration of androgen hormones [207]. However, this evidence is limited to preclinical studies as improvements seemed to be poor compared to the systemic adverse effects of treating females with androgen hormone therapy.

#### 6.3.2. Lentiviral-Mediated Gene Therapy in Pompe Disease

Ex vivo studies have applied hematopoietic stem cell (HSC) gene therapy. It consists of isolating CD34+ cells from mobilized peripheral blood and transducing them with a lentiviral vector containing the functional *GAA* gene [208]. Then, the modified cells are infused back into the patient who was previously conditioned with alkylating agents, such as busulfan, in order to achieve repopulation of the bone marrow with stem cells capable of secreting functional GAA enzyme [208]. The greatest advantages of HSC gene therapy are the possibility of single administration and the immune tolerance induction that has already been demonstrated after HSC gene transfer of other exogenous proteins. The first trial evaluating this approach was published in 2009 [209]. A partial restoration of GAA enzymatic activity was observed in bone marrow and peripheral blood cells, with less efficacy in reducing glycogen storage in the heart [209].

By using an LV vector having a stable mammalian promoter and a human beta-globin locus control region, PD mice showed great improvement in hypertrophic cardiomyopathy and an increase in motor function, although not statistically significant compared to untreated mice 6 months post-transplant [210]. LV gene therapy in murine models also seems to be effective in CNS involvement. Transplantation of HSCs expressing codon-optimized *GAA* reduced glycogen to near normal levels in brain tissue [211]. In this study, immunofluorescence staining of mouse brain sections 3.5 months post-transplantation showed GAA expression in a large proportion of microglia and virtually all astrocytes, probably because of their expression of M6P receptors [211].

### 6.4. Other Experimental Therapies

Additional experimental therapies have targeted oxidative stress, splicing dysfunction, and substrate reduction.

As oxidative stress is linked to dysfunctional autophagy in PD pathogenesis, a pre-clinical study recently evaluated the association between ERT and antioxidant agents, suggesting that they may improve GAA activity in rhGAA-treated cells [212]. The use of splice-switching antisense oligonucleotides (AOs) has also been investigated. Most AO studies have focused on the common c.-32-13T>G mutation known to cause skipping of exon 2 from *GAA* transcripts [213]. This exon contains the translation start codon, and its skipping promotes mRNA degradation [213]. In this case, the function of AOs may be to modulate splicing by blocking splicing regulatory sequences. The feasibility of AO-mediated exon correction was explored by Van der Wal et al. in Pompe patient-derived myotubes and fibroblasts, and an increase in GAA enzyme activity was observed above the 20% threshold [214]. A recent study showed dose-dependent improvement in GAA activity in myogenic differentiation 1-induced myogenic cells derived from nine patients with LOPD, although the responses were influenced by cell quality and the nature of the second mutation [215]. Evaluating response in clinical trials is necessary as genomic sequence homology in the exon 2 region of *GAA/Gaa* between humans and mice is only around 50% [215]. A substrate reduction-based therapy inhibiting glycogen synthesis could be used as an adjunctive strategy to ERT. Antisense oligonucleotide-induced exon 6 skipping of the glycogen synthase 1 (Gys1) gene, which encodes skeletal muscle glycogen synthase activity, has shown great reduction in skeletal enzyme levels in an animal model [216]. More recently, the development of small-molecule inhibitors of human skeletal muscle glycogen synthase, such as substituted pyrazoles, has led to a phase 1 clinical trial in healthy individuals (NCT05249621) [217].

## 7. Conclusions

The diagnosis of PD has considerably improved over the years thanks to increased awareness among clinicians, the spread of newborn screening and clinical use of DBS, and the application of novel diagnostic tools, such as NGS and muscle MRI. An interesting point of discussion is the best outcome measures that future clinical trials for PD should evaluate. In most studies, FVC and 6MWT are set as the primary outcomes, even though they have some limitations. The evaluation of novel biomarkers is needed to assess the progression of the disease and guide clinicians in the timing of treatment. ERT represents the first and only treatment approved for PD. Although the advent of ERT has changed the natural history of the disease and the quality of life of patients, many unresolved issues remain, and limitations of treatment include the variable response and reduced duration of efficacy, which tends to decrease after the first few years of treatment. Next-generation ERT could improve this trend and have longer lasting efficacy than the current recombinant enzyme. Since the timing of treatment may influence the response to therapy, ERT should be started early when clinical, pathological, or MRI signs of disease appear. The wide accessibility of newborn screening for PD around the world also raises the question of when to start treatment in those subjects who are found to carry LOPD-associated variants and may develop clinical signs of disease much later in life. This will be a pressing problem in the near future and clinicians will have to confront each other to address it unambiguously.

More recently, the application of gene therapy has been explored. Even though gene therapy has the advantage of single administration of treatment, more studies are required to assess its efficacy in glycogen clearance in different tissues and clinical response. A critical discussion of all of these issues is crucial when looking forward to the future prospects of more effective treatments that would further change the natural history of the disease.

## Figures and Tables

**Table 2:** Some of the most common variants in the *GAA* gene.

DNA Nomenclature	Site	Protein Nomenclature	Type of Variant	Biochemical Evidence	Predicted Severity	Predicted Phenotype with Null Allele	Population	GnomAD Frequency
c.-32-13T>G	Intron 1	p.[=,0]	Deletion (translation initiation site) No effect on splicing	Protein is expressed Variant causes skipping of exon 2	Potentially mild	LOPD (childhood or adult onset)	Caucasian	0.00309
c.2560C>T	Exon 18	p.(Arg854*)	Non-sense	No protein expression	Very severe	IOPD	African Descendent	0.00048
c.841C>T	Exon 4	p.(Arg281Trp)	Missense	Protein is expressed	Potentially mild	LOPD	European (non-Finnish)	0.00033
c.525del	Exon 2	p.(Glu176Argfs*45)	Frameshift	No protein expression	Very severe	IOPD	European (non-Finnish)	0.00013
c.1979G>A	Exon 14	p.(Arg660His)	Missense	Protein is expressed	Potentially mild	LOPD (childhood onset)	African	0.00009
c.853C>T	Exon 4	p.(Pro285Ser)	Missense	Protein is expressed	Mild	LOPD	African	0.00007
c.655G>A	Exon 3	p.(Gly219Arg)	Missense	Protein is expressed	Potentially mild	IOPD	African	0.00006
c.953T>C	Exon 5	p.(Met318Thr)	Missense	Protein is expressed	Potentially mild	IOPD	African	0.00006
c.1935C>A	Exon 14	p.(Asp645Glu)	Missense	Protein is expressed	Potentially mild	IOPD	South Asian	0.00004

From: https://clinvarminer.genetics.utah.edu/variants-by-gene/GAA/condition/Glycogen%20storage%20disease%2C%20type%20II/pathogenic (accessed on 1 May 2023).

**Table 3:** Main clinical findings in LOPD.

	LOPD
Clinical Examination	Limb-girdle and axial weakness Tongue involvement Respiratory insufficiency Easily fatigued
Systemic Involvement	Musculoskeletal complications (i.e., hyperlordosis, kyphosis, scoliosis, spine rigid syndrome) Brain vascular abnormalities
Laboratory Tests	Mildly elevated or normal serum creatine kinase levels Elevation of AST/ALT levels
EMG	Myopathic pattern with spontaneous activity including myotonic discharges, especially in axial muscles
Muscle MRI	Paravertebral and abdominal muscle involvement Tongue involvement Hip extensor involvement (i.e., adductor magnus) with sparing of leg muscles
Muscle Biopsy	Vacuolar myopathy with glycogen storage and acid phosphatase reactivity

**Table 4:** Main current and future therapeutic approaches for Pompe disease.

Therapy	Mechanism of Action	Status of Approval
*Alglucosidase alfa*	Enzyme replacement therapy by intravenous infusion of recombinant human GAA (rhGAA)	First approved 28 April 2006 (FDA)
*Avalglucosidase alfa*	Enzyme replacement therapy by intravenous infusion of recombinant human GAA (rhGAA)	First approved 6 August 2021 (FDA) *Active study recruiting:* An Open-label, Multinational, Multicenter, Intravenous Infusion Study of the Efficacy, Safety, Pharmacokinetics, and Pharmacodynamics of Avalglucosidase Alfa in Treatment Naïve Pediatric Participants With Infantile-Onset Pompe Disease (IOPD)
*Cipaglucosidase alfa +* *miglustat*	Enzyme replacement therapy by intravenous infusion of recombinant human GAA (rhGAA) + small-molecule ligands (chaperones) as enzyme stabilizers	First approved 23 March 2023 for LOPD (EU) *Active study recruiting:* Open-label Study to Evaluate the Safety, Efficacy, Pharmacokinetics, Pharmacodynamics, and Immunogenicity of Cipaglucosidase Alfa/Miglustat in Both ERT-Experienced and ERT- Naïve Pediatric Subjects With Infantile-Onset Pompe Disease Aged 0 to < 18 Years Open-label Study of the Safety, Pharmacokinetics, Efficacy, Pharmacodynamics, and Immunogenicity of Cipaglucosidase Alfa/Miglustat in Pediatric Subjects Aged 0 to < 18 Years With Late- Onset Pompe Disease
*Gene Therapy*	Providing cells with a healthy copy of the *GAA* gene to restore functional GAA enzyme production	Not approved. *Active study recruiting:* Single Arm, Multicenter, Open and Dose-Escalation Clinical Study on Safety, Tolerance, and Efficacy of GC301, an AAV-Delivered Gene Transfer Therapy in Patients with Infantile-Onset Pompe Disease *Active study, not recruiting*: Phase 1 Study of the Safety of AAV2/8-LSPhGAA (ACTUS-101) in Late-Onset Pompe Disease Phase 1/2, Dose-Escalation Study to Evaluate the Safety, Tolerability, and Efficacy of a Single Intravenous Infusion of SPK-3006 in Adults with Late-Onset Pompe Disease Open-Label, Fixed-Sequence, Ascending-Dose, First-in-Human Study to Assess the Safety, Tolerability, Pharmacokinetics, Pharmacodynamics, and Efficacy of Intravenous Infusions of ATB200 Co-Administered with Oral AT2221 in Adult Subjects with Pompe Disease Phase 3 Open-label Extension Study to Assess the Long-Term Safety and Efficacy of Intravenous ATB200 Co-Administered With Oral AT2221 in Adult Subjects with Late-Onset Pompe Disease
*Substrate Reduction Therapy*	Small-molecule inhibitors of human skeletal muscle glycogen synthase	Not approved. *Active study, not recruiting:* Phase 1, Randomized, Double-Blind, Placebo-Controlled, Single and Multiple Ascending Dose Study of MZE001 to Evaluate the Safety, Tolerability, Pharmacokinetics, and Pharmacodynamics in Healthy Subjects
